# Transcriptome Analysis to Identify the Putative Biosynthesis and Transport Genes Associated with the Medicinal Components of *Achyranthes bidentata* Bl.

**DOI:** 10.3389/fpls.2016.01860

**Published:** 2016-12-12

**Authors:** Jinting Li, Can Wang, Xueping Han, Wanzhen Qi, Yanqiong Chen, Taixia Wang, Yi Zheng, Xiting Zhao

**Affiliations:** ^1^College of Life Sciences, Henan Normal UniversityXinxiang, China; ^2^Engineering Laboratory of Biotechnology for Green Medicinal Plant of Henan ProvinceXinxiang, China; ^3^Boyce Thompson Institute, IthacaNY, USA

**Keywords:** *Achyranthes bidentata* Bl., RNA-Seq, oleanolic acid, ecdysterone, MVA (mevalonic acid) pathway

## Abstract

*Achyranthes bidentata* is a popular perennial medicine herb used for 1000s of years in China to treat various diseases. Although this herb has multiple pharmaceutical purposes in China, no transcriptomic information has been reported for this species. In addition, the understanding of several key pathways and enzymes involved in the biosynthesis of oleanolic acid and ecdysterone, two pharmacologically active classes of metabolites and major chemical constituents of *A. bidentata* root extracts, is limited. The aim of the present study was to characterize the transcriptome profile of the roots and leaves of *A. bidentata* to uncover the biosynthetic and transport mechanisms of the active components. In this study, we identified 100,987 transcripts, with an average length of 1146.8 base pairs. A total of 31,634 (31.33%) unigenes were annotated, and 12,762 unigenes were mapped to 303 pathways according to the Kyoto Encyclopedia of Genes and Genomes pathway database. Moreover, we identified a total of 260 oleanolic acid and ecdysterone genes encoding biosynthetic enzymes. Furthermore, the key enzymes involved in the oleanolic acid and ecdysterone synthesis pathways were analyzed using quantitative real-time polymerase chain reaction, revealing that the roots expressed these enzymes to a greater extent than the leaves. In addition, we identified 85 ATP-binding cassette transporters, some of which might be involved in the translocation of secondary metabolites.

## Introduction

*Achyranthes bidentata* Bl., a member of Amaranthaceae, is an erect perennial herbaceous plant widely distributed and grown in Asian countries, including China, India, Korea, and Japan. In China, this species is primarily distributed in the Guhuaiqingfu area of Henan Province. The dried roots of *A. bidentata* have been prescribed in the Chinese Pharmacopeia as an important herbal medicine with multiple pharmacological effects for the treatment of lumbar and knee osteodynia, muscle spasms and limb flaccidity. Pharmacological studies have revealed that this crude drug can relieve pain, diminish inflammation, excite uterine contractions, decrease blood glucose, reduce blood fat, reinforce immunological function, delay senility, exhibit anti-oxidant capacity and confer cardio protection (Wei et al., 2012; Zhang M. et al., 2012; Tie et al., 2013). It has been reported that *A. bidentata* could significantly prevent osteoblast damage and bone deterioration (Jiang et al., 2014; Suh et al., 2014). Similarly, it has been shown that 16 weeks of AE (*A. bidentata* root extract) treatment improved bone biomechanical quality through modifications of bone mineral density and trabecular microarchitecture without a hyperplastic effect on uterine ovariectomy-induced osteoporosis in rats (Zhang R. et al., 2012). The main ingredients of AE are triterpenoid saponins with oleanolic acid aglycon, ecdysterone, and polysaccharide (Li et al., 2005).

Triterpenoid saponins and phytoecdysteroids are widely distributed in higher plants and exhibit important activities, depending on their structure. Triterpenoid saponins are enriched in the roots of *A. bidentata*, and it has been reported that oleanolic acid glycosides may be the active components in this herb for the treatment of osteoporosis (Li et al., 2005). Phytoecdysteroids, plant-derived analogs of insect molting hormones, have attracted much research attention for nearly 50 years. The naturally occurring ecdysteroid hormone 20-hydroxyecdysone (20E) is the most abundant phytoecdysteroid with relative levels in various organs of *A. bidentata* (fruits > leaves > roots > stems) (Li et al., 2007). In addition, the experiments demonstrated that ecdysterone from *A. bidentata* increased osteoblastic activity and protected chondrocytes (Gao et al., 2000; Zhang et al., 2014).

Despite the economic and pharmacological value of *A. bidentata*, there is no genetic information revealing the biosynthesis of these active constituents. Recent advances in transcriptome deep-sequencing technology and bioinformatics have opened a new avenue for such investigations (Hwang et al., 2015; Zhang et al., 2015; Rai et al., 2016). The transcriptomes of various plants have previously been investigated, e.g., *Polygonum quinquefolium* (Sun et al., 2010), *Lolium multiflorum* (Pan et al., 2016), *Ipomoea batatas* (Ma et al., 2016), *Chrysanthemum morifolium* (Xu et al., 2013), and *Juglans mandshurica* (Hu et al., 2016). However, genomic studies of *A. bidentata* have not yet been reported. Limited transcriptome studies on *A. bidentata* had been conducted by Li et al. (2015). They studied the development of microsatellite markers for *A. bidentata* using 454 pyrosequencing combined with magnetic bead enrichment. Meanwhile, they have obtained a total of 903 microsatellite loci from 42,004 individual sequence reads. This set of markers will provide useful tools for examining genetic diversity and population structure, and aid in better understanding of the conservation of *A. bidentata*. However, there are no report that provided valuable insights to the identification of the conserved metabolic pathways of medicinal components in *A. bidentata*, especially for oleanane-type saponin and ecdysterone biosynthesis pathways.

Leaves and roots, both as the key parts in plant, have distinct functions. Leaves are mainly in charge of converting light into chemical energy and accumulating assimilates through photosynthesis, while roots serve as the major storage of energy (e.g., polysaccharide) and absorb water and mineral nutrients. The botanical, physiological, genetic and secondary metabolism processes in the leaves and roots have consistently drawn considerable research attention. However, the molecular mechanisms of the biosynthesis and transport of medicinal ingredients between the leaf and root are still unknown. These indicate that the biosynthetic relationships and physiological coordination between them are complex. The results of our former experiments demonstrated that the oleanolic acid content of *A. bidentata* root was the highest at the vegetative growth period and amount to 7.76% (Li and Hu, 2009). Similarly, our former studies also suggested that at the vegetative growth stage, ecdysterone content from leaves of *A. bidentata* was the highest (Li et al., 2007). In addition, with the development process, the amount of oleanolic acid in roots increased gradually again and tended to stabilize after reaching 2.95% in October, while the amount of oleanolic acid in stems and leaves began to decrease gradually. This is most likely because of medicinal ingredients from leaf to root through transporters. However, there is no report about transporters in *A. bidentata*. So we selected leaves and roots of *A. bidentata* at the vegetative growth period as material in order to understand of several key pathways and enzymes involved in the biosynthesis and transport of oleanolic acid and ecdysterone. In the present study, Illumina deep RNA sequencing (HiSeq 2500, Illumina, Shanghai) was used to annotate and analyze the leaf and root transcriptomes of young *A. bidentata*. Putative genes involved in the biosynthetic pathway of triterpenoid saponins and phytoecdysteroid and modification of backbone were identified. As we know, this study reported the *do novo* sequencing of *A. bidentata* for the first time. Meanwhile, crucial genes regulating different metabolic pathways, especially the ones for biosynthesis and transportation of putative medicinally important secondary metabolites, were identified by transcriptome analysis. Furthermore, it provides valuable information for developing important molecular markers, such as sequence repeats (SSRs), to enhance the medicinal traits of *A. bidentata*.

## Materials and Methods

### Plant Materials

**FIGURE 1:**
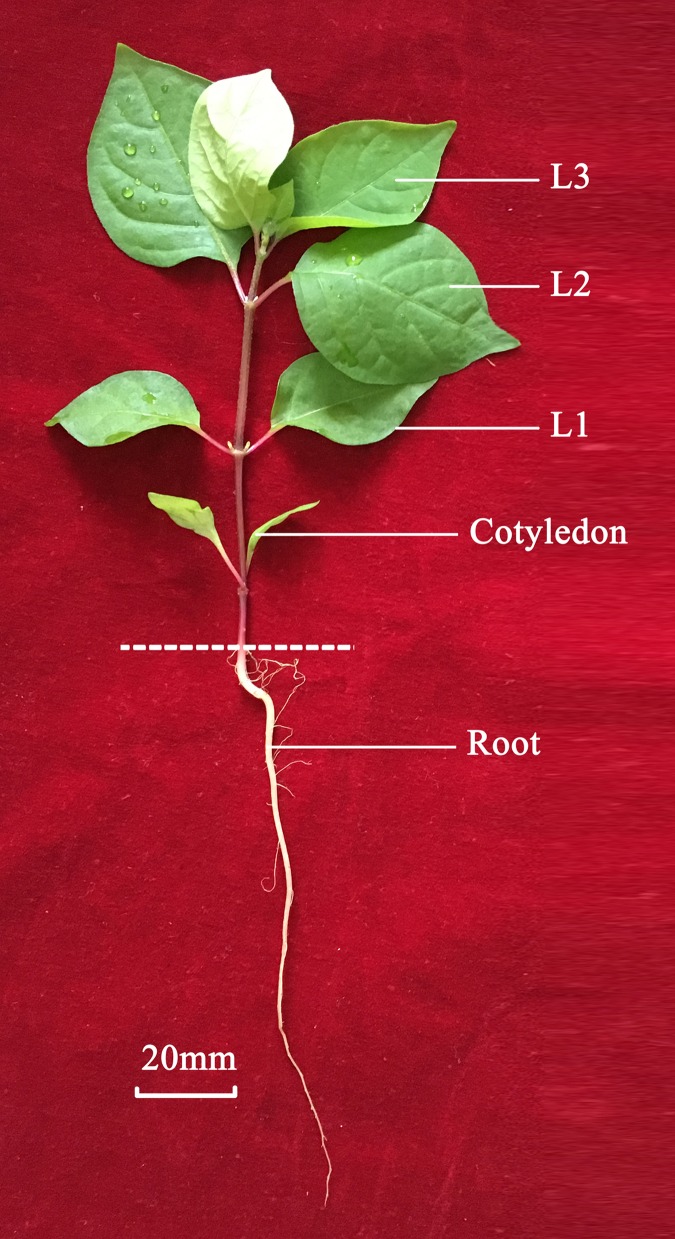
**Root and leaf of *Achyranthes bidentata* used for transcriptome study**.

*Achyranthes bidentata* Blume was collected from the Wenxian Agricultural Science Institute of Henan Province in China. The leaves (L2 and L3) and roots were dissected from the plants, immediately frozen in liquid nitrogen, and stored at -80°C (**Figure 1**). Three biological replicates were used for RNA extraction and two replicates for transcriptome sequencing of the leaf and root.

### Total RNA Extraction and cDNA Library Construction for Sequencing

Total RNA was isolated from the leaves and roots using RNAqueous^TM^ phenol-free total RNA (Austin, TX, USA) according to the manufacturer’s instructions. The yield and purity of RNA were determined using a NanoDrop 2000 spectrophotometer and Agilent Bioanalyzer 2100 (Agilent Technologies, USA) with a 28S:18S greater than 1.5 and RIN (RNA integrity number) greater than 7. Qualified total RNA was further purified using the RNeasy micro kit (QIAGEN, GmBH, Germany) and RNase-Free DNase I (QIAGEN) and subsequently used in applied to cDNA library construction and Illumina deep sequencing.

Illumina sequencing: mRNAs with poly(A) tails were extracted from total RNA by beads with oligo (dT), then sheared into small pieces with Fragment Mix. Double-stranded cDNA was synthesized from cleaved mRNA fragments. The short cDNA fragments were purified using Agencourt^®^ AMPure XP Beads (Beckman Coulter, USA) and resolved with 80% ethanol for end reparation and poly(A) addition. Subsequently, the short cDNA fragments were attached with sequencing adapters using DNA Ligase Mix, RNA Adapter Index and Stop Ligase Mix. Polymerase chain reaction (PCR) amplification: electrophoresis was performed to select the appropriate fragments as templates. Two cDNA libraries for leaves and two for roots were sequenced on the platform of Illumina HiSeq 2500 to generate paired-end reads.

### *De novo* Transcriptome Assembly

Clean data (clean reads) were screened from raw sequencing reads after the removal of contaminated reads, including adapter sequences, reads containing poly-N, reads shorter than 20 nt, ribosome RNA reads, and low-quality sequencing reads. In addition, the Q20 and Q30 values, the GC-content, and the sequence duplication level of the clean data were calculated. *De novo* assembly was performed using scaffolding contigs in CLC Genomics Workbench (version 6.0.4), with the parameters word-size = 45 and minimum contig length ≥ 300 (Bräutigam et al., 2011; Garg et al., 2011; Su et al., 2011). CAP3 EST stitching software was employed for primary UniGene second splicing to render a final unigene set of sequences. All downstream analyses were based on high-quality clean data.

### Functional Annotation and Classification of Unigenes

After sequence assembly, the unigene sequences were aligned using BLASTX to protein databases, such as Swiss-Prot, Kyoto Encyclopedia of Genes and Genomes (KEGG) and COG, to retrieve proteins with the highest sequence similarity to the given unigenes and putative functional annotations. Gene ontology (GO) terms were assigned to the *A. bidentata* assembled transcripts based on the GO terms annotated to the corresponding homologs in the UniProt database. Subsequently, the GO annotation of unigenes was realized according to molecular function, biological processes and cellular component (Conesa et al., 2005; Ye et al., 2006). The COG database was applied to classify orthologous gene products. Therefore, we aligned unigenes to the COG database to classify and predict the possible functions of the unigenes. The KEGG database is typically used to analyze gene products during the metabolism process and gene function in the cellular processes. Thus, biochemical pathways were predicted from the *A. bidentata* transcripts using the KEGG Automatic Annotation Server (KAAS) pathway alignment analysis tools online.

### Differential Gene Expression and Pathway Analysis

The high-quality cleaned RNA-Seq reads were aligned to the assembled *A. bidentata* transcripts. Following alignment, raw counts for each *A. bidentata* transcript in each sample were derived and normalized to reads per kilobase of exon model per million mapped reads (RPKM). The *P*-value sets the threshold for differential gene expression tests. Differentially expressed genes (DEGs) (fold-changes ≥ 2 or fold-changes ≤ 0.5 with *P*-value < 0.05) between the roots and leaves were determined as up-regulated or down-regulated using the DESeq R package.

Gene ontology functional and KEGG Pathway enrichment analyses were conducted for the DEGs. First, all DEGs were BLASTed in the GO database^1^ and the gene numbers were calculated for each GO term using GO-Term Finder v. 0.86^2^ (Boyle et al., 2004). Subsequently, a hypergeometric test was used to identify significantly enriched GO terms in DEGs for comparison with the genome background based on the *P-*value. The calculated *P-*value was calibrated using Bonferroni’s correction. GO terms were defined as significantly enriched in DEGs when the corrected *P* ≤ 0.05. The pathway enrichment analysis identifies significantly enriched metabolic pathways or signal transduction pathways in DEGs compared with the entire genome background. The calculated *P-*value for the pathway enrichment analysis was similar to that for the GO analysis. After multiple testing corrections, the pathways with a *P* ≤ 0.05 were considered significantly enriched in DEGs.

### Real-Time Quantitative Reverse Transcription Polymerase Chain Reaction (qRT-PCR) Validation and Expression Analysis

Seventeen DEGs involved in the triterpenoid saponin and ecdysterone metabolic pathways were chosen for verification of Illumina RNA-Seq results using real-time qPCR. The gene-specific primers, designed in Primer Premier 5.0 software, are detailed in Supplementary Table S1. All reactions were performed in 96-well plates in a LightCycler 96 (Roche, Switzerland) using EvaGreen 2X qPCR Master Mix (ABM, Canada) according to the manufacturer’s instruction. All reactions were assayed in biological triplicates with three technical replicates per experiment, and the results were expressed relative to the expression levels of an internal reference gene, *Ubiquitin* (UN008343), in each sample using the 2^-ΔΔCt^ method.

## Results

### Sequencing and *De novo* Assembly of the *A. bidentata* Transcriptome

We first calculated correlation coefficients of transcriptome profiles among the four samples and between the technical replicates (**Table 1**). The high correlation among biological replicates and between technique replicates indicated the robustness of our RNA-seq dataset. In this study, we obtained 35,359,331, 27,630,495, 34,923,635, and 33,637,585 Illumina raw reads, respectively, for the two leaf samples and two root samples, generating 3.53 G, 2.76 G, 3.49 G, and 3.36 G total bases, respectively (**Table 2**). After eliminating the primer and adapter sequences and filtering out the low-quality reads, we pooled all the high-quality Illumina reads from the two libraries. Subsequently, we combined all reads to build a transcriptome database for *A. bidentata* Bl. We identified 100,987 transcripts with a total of 115,810,705 bp. The mean size of each transcript was 1146.8 bp, and the maximum length sequence was 17,205 bp (**Table 2**).

**Table 1:** Correlation coefficients of transcriptome profiles among RNA-seq samples.

	Ab_L_rep1	Ab_L_rep2	Ab_R_rep1	Ab_R_rep2
Ab_L_rep1	1	0.99	0.15	0.14
Ab_L_rep2	0.99	1	0.11	0.11
Ab_R_rep1	0.15	0.11	1	0.99
Ab_R_rep2	0.14	0.11	0.99	1


*Ab_L_rep1, Ab_L_rep2: leaves samples.*

*Ab_R_rep1, Ab_R_rep2: roots samples.*

**Table 2:** Summary of Illumina transcriptome sequencing for leaves and roots of *Achyranthes bidentata*.

	Ab-L		Ab-R	
	
	Ab-L-rep1	Ab-L-rep2	Ab-R-rep1	Ab-R-rep2
Raw reads	35,359,331	27,630,495	34,923,635	33,637,585
Clean reads	29,637,493	22,759,674	29,470,859	28,248,696
No. of mapped read pairs		87,242,692		
Number of assembly transcripts (n)		100,987		
Average length (bp)		1,146.8		
Total length of transcripts (bp)		17,205		
Min length (bp)		224		
Total length of transcripts (bp)		115,810,705		


### Annotation of *A. bidentata* Unique Transcript Sequences

**FIGURE 2:**
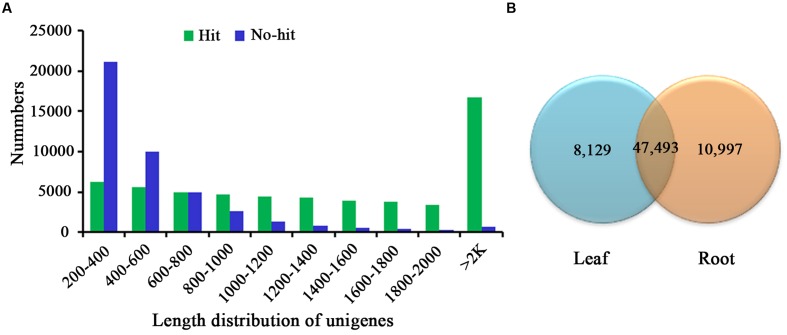
**Comparison of unigene length between hit and no-hit in UniProt database **(A)**.** The Venn diagram shows the number of expressed genes (RPKM ≥ 1) in samples of leaf and root **(B)**.

**FIGURE 3:**
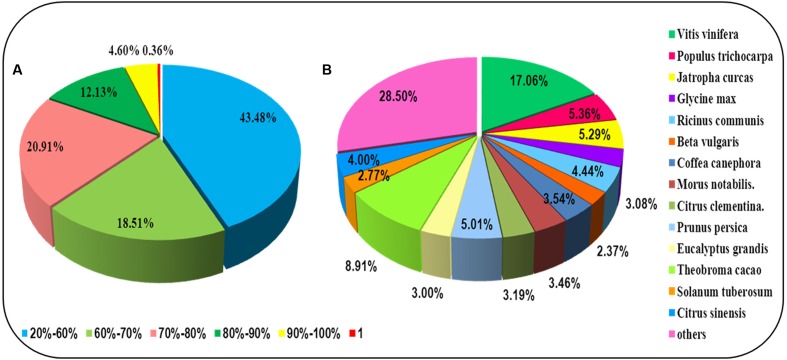
**Results summary for sequence-homology searching against the UniProt database.** **(A)** Similarity distribution of the closest BLASTX matches for each sequence. **(B)** A species-based distribution of BLASTX matches for sequences. We used all plant proteins in the UniProt database in performing the homology search.

For annotation, the sequences were searched against the Swiss-Prot database using BlastX (Altschul et al., 1997) with a cut-off E-value of 1e-5. 31,634 unigenes in total indicated significant similarity to known proteins in the Swiss-Prot database. The unigenes length distribution of the hit and no-hit is shown in **Figure 2A**, revealing that longer contigs most likely represented Blast matches in the proteins databases (Wang et al., 2010). The results also indicated that 79.8% of unigenes over 600 bp in length had Blast matches in the Swiss-Prot database, while only 23.0% of unigenes shorter than 400 bp had Blast matches in the Swiss-Prot database (**Figure 2A**). A Venn diagram of the genes expressed with reads per kilobase of transcript per million mapped reads (RPKM) ≥ 1 is demonstrated in **Figure 2B**. A total of 47,493 unigenes were expressed in both the samples of leaf and root in *A. bidentata*. The results of similarity distribution indicated that 17.09% of the matches were of high similarity (≥80% similarity in BlastX results), while 39.42% were within 60–80% (**Figure 3A**). Meanwhile, the matching sequence analysis showed the closest matches to the sequences of *Vitis vinifera* (17.06%), the sequences of *Theobroma cacao* (8.91%) and the sequences of *Populus trichocarpa* (5.36%) (**Figure 3B**). Thus, *A. bidentata* has a close genetic relationship with *Vitis vinifera*.

### GO (Gene Ontology) Functional Classification

**FIGURE 4:**
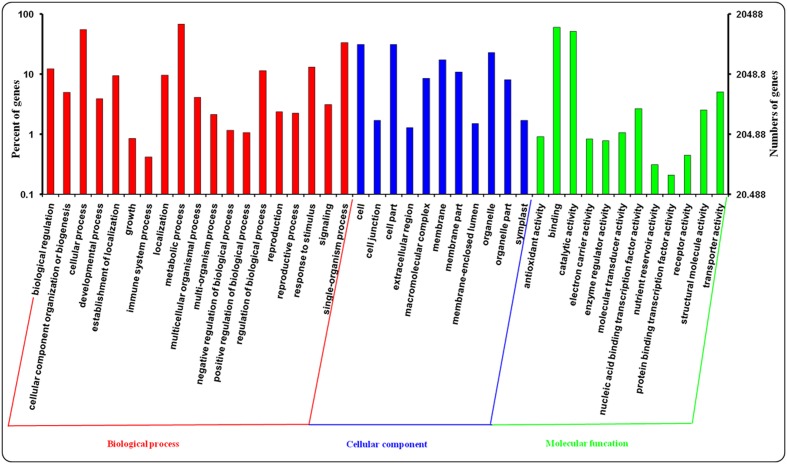
**Gene ontology (GO) assignments for leaves and roots transcriptome of *A. bidentata***.

Gene ontology assignments were applied to classify the predicted function of leaf and root genes in *A. bidentata*. In our study, *A. bidentata* Bl. genes were classified into ‘Biological process,’ ‘Cellular component,’ and ‘Molecular function’ GO categories and further sub-divided into 42 sub-categories. Among the 100,987 assembled transcripts, 20,488 transcripts were successfully annotated with GO assignments; some of these transcripts belonged to one or more of the three categories (**Figure 4**). The ‘Biological process’ sub-categories included metabolic process (14,053, 68.59%), cellular process (11,404, 55.66%), single-organism process (6,811, 33.24%), response to stimulus (2,705, 13.20%), biological regulation (2,500, 12.20%), regulation of biological process (2,346, 11.45%) and other sub-categories (9,292, 45.35%). The major proportion of the ‘Cellular component’ sub-categories included cell (6,407, 31.27%), cell part (6,407, 31.27%), organelle (4,705, 22.96%), membrane (3,566, 17.41%), membrane part (2,220, 10.84%), macromolecular complex (1,741, 8.50%), organelle part (1,662, 8.11%), and other sub-categories (1,265, 6.17%). The top three most abundant sub-categories of the ‘Molecular function’ category were binding (12,389, 60.47%), catalytic activity (10,625, 51.86%), and transporter activity (1,037, 5.06%) (Supplementary Table S2).

### COG (Clusters of Orthologous Groups) Functional Classification

**FIGURE 5:**
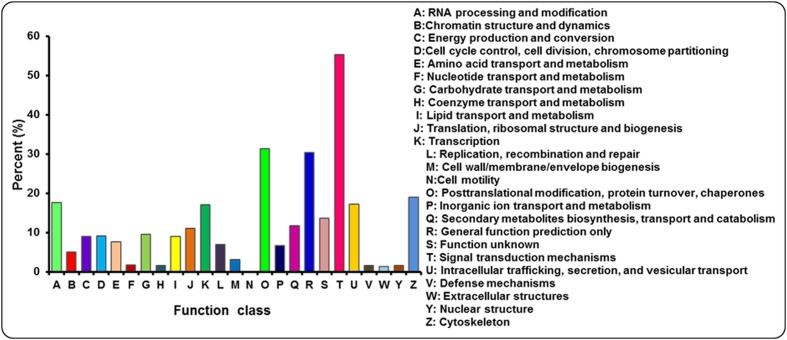
**COG functional classification for leaves and roots transcriptome of *A. bidentata***.

The annotated sequences searched against the COG database for functional prediction and classification. Based on sequence homology, 21,715 unigenes were classified into 25 COG categories involved in signal transduction, cellular process, metabolism and other processes (**Figure 5**; Supplementary Table S3). The largest category was signal transduction mechanisms (12,007, 55.29%), followed by post-translational modification; protein turnover and chaperones (6800, 31.31%), general function prediction only (6604, 30.41%), cytoskeleton (4119, 18.97%) and RNA processing and modification (3842, 17.69%). Only a few unigenes were assigned to extracellular structures (283, 1.30%) and cell motility (8, 0.04%). In addition, 2059 and 1441 unigenes were clustered into carbohydrate transport and metabolism and inorangic ion transport and metabolism, respectively. In addition, it is interesting to note that 2550 unigenes represented the secondary metabolites biosynthesis, transport and catabolism category, indicating a lot of secondary metabolites present in *A. bidentata*.

### KEGG (Kyoto Encyclopedia of Genes and Genomes) Pathway Annotation

**FIGURE 6:**
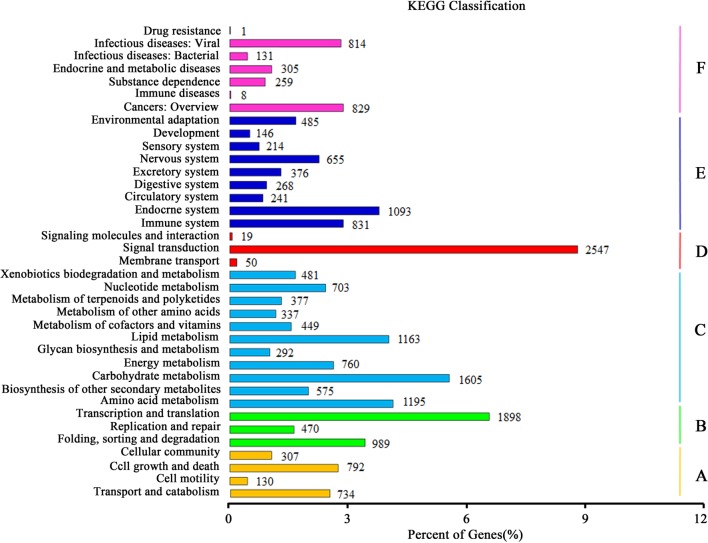
**Kyoto encyclopedia of genes and genomes (KEGG) classification of unigenes.** **(A)** Cellular Processes; **(B)** Genetic Information Processing; **(C)** Metabolism; **(D)** Environmental Information Processing; **(E)** Organismal Systems; **(F)** Human Diseases.

Kyoto Encyclopedia of Genes and Genomes pathway analysis was fulfilled to identify the active biological pathways in the root and leaf of *A. bidentata*. In total, 12,762 unigenes with significant matches in the database were assigned to 303 pathways (Supplementary Table S4). Unigenes classified to the six main KEGG biochemical pathways, cellular process, genetic information processing, metabolism, environmental information processing, organismal systems and human diseases are shown in **Figure 6**. In the metabolism category, the maximum number of unigenes fell under carbohydrate metabolism (1605 unigenes) followed by amino acid metabolism (1195 unigenes) and lipid metabolism (1163 unigenes). We also obtained 377 genes involved in the metabolism of terpenoids and polyketides, including terpenoid backbone biosynthesis (86 unigenes), sesquiterpenoid and triterpenoid biosynthesis (22 unigenes), monoterpenoid biosynthesis (18 unigenes), carotenoid biosynthesis (48 unigenes), and diterpenoid biosynthesis (26 unigenes). Many enzymes mapped to unigenes in KEGG pathways indicate that the active metabolic processes were underway in the leaves and roots of *A. bidentata*, and a variety of metabolites were synthesized.

### Identification and Functional Classification of Differentially Expressed Genes

To identify different expression levels of unigenes between roots and leaves of *A. bidentata*, we calculated the RPKM values of assembled unigenes. The total number of DEGs between roots and leaves was 28,339, with an adjusted *P* < 0.05 as the threshold. In roots vs. leaves, 15,580 genes were up-regulated, and 12,759 genes were down-regulated.

Differentially expressed genes of from the DGE analysis were further analyzed using GO and KEGG enrichment to determine their potential function and within a metabolic pathway. The GO classification of DEGs between the different tissues is shown in Supplementary Table S5. GO enrichment analysis of the DEGs in root vs. leaf showed that they were enriched in 44 subcategories, with cell (GO:0005623; 16,182 DEGs), cell part (GO:0044464; 16161 DEGs) and intracellular (GO:0005622; 13,476 DEGs) representing the most abundant ‘Cell component’ category. In the ‘Biological process’ category, the top three enriched terms were cellular process (GO:0009987, 144,745 DEGs), single-organism process (GO:0044699, 13,476 DEGs) and metabolism (GO:0008152, 12,788 DEGs). The major proportion of the ‘Molecular function’ category included binding (GO: 0044699, 12,123 DEGs) and catalytic activity (GO:0044699, 10,776 DEGs). In addition, it was interesting note that we also identified 915 and 566 DEGs that participated in secondary metabolic process and terpenoid biosynthetic process, respectively.

Further identification of biosynthetic as well as other important pathways was implemented in the KEGG database, as to advance the exploration of DEGs functions in detail. The significant pathways (corrected *P* < 0.05) identified in both root vs. leaf were involved in the biosynthesis of saponin and its derivatives, including farnesene biosynthesis, superpathway of geranylgeranyl diphosphate biosynthesis I (via mevalonate), superpathway of linalool biosynthesis, and *trans*-lycopene biosynthesis II (plants). In addition, C4 photosynthetic carbon assimilation cycle, NAD-ME type, C4 photosynthetic carbon assimilation cycle, PEPCK type, oxygenic photosynthesis, photosynthesis light reactions and chlorophyll cycle were significantly enriched pathways among the DEGs between the different tissues. These findings are consistent with the functions of leaf photosynthesis. Moreover, we also identified genes involved in the degradation of secondary metabolites, such as betanidin, baicalein, and luteolin triglucuronide (**Table 3**).

**Table 3:** Kyoto Encyclopedia of Genes and Genomes (KEGG) functional classification for leaves and roots transcriptome of *A. bidentata*.

Pathway	Pathway ID	Nd	Bn	*P*-value	adj P
Farnesene biosynthesis	PWY-5725	8	8	9.77E-04	1.72E-02
*trans*-farnesyl diphosphate biosynthesis	PWY-5123	33	41	5.00E-07	2.63E-05
Superpathway of geranylgeranyldiphosphate biosynthesis I (via mevalonate)	PWY-5910	48	61	4.55E-09	4.20E-07
Geranyl diphosphate biosynthesis	PWY-5122	30	36	4.00E-07	2.46E-05
Superpathway of linalool biosynthesis	PWY2OL-4	39	45	6.02E-10	2.22E-07
Linalool biosynthesis I	PWY-7182	38	44	1.26E-09	2.32E-07
Linalool biosynthesis II	PWY-7141	31	37	1.98E-07	1.46E-05
β-caryophyllene biosynthesis	PWY-6275	8	8	9.77E-04	1.72E-02
*trans*-lycopene biosynthesis II (plants)	PWY-6475	24	33	3.41E-04	7.86E-03
Superpathway of scopolin and esculin biosynthesis	PWY-7186	11	13	2.14E-03	3.43E-02
C4 photosynthetic carbon assimilation cycle, NAD-ME type	PWY-7115	42	60	9.84E-06	3.63E-04
C4 photosynthetic carbon assimilation cycle, PEPCK type	PWY-7117	36	52	6.28E-05	2.11E-03
Oxygenic photosynthesis	PHOTOALL-PWY	42	64	1.09E-04	2.68E-03
Photosynthesis light reactions	PWY-101	12	15	3.19E-03	4.91E-02
Chlorophyll cycle	PWY-5068	9	9	4.10E-04	8.40E-03
Cyclopropane fatty acid (CFA) biosynthesis	PWY0-541	26	27	2.42E-09	2.97E-07
Alkane oxidation	PWY-2724	18	19	1.86E-06	8.60E-05
UDP-D-xylose biosynthesis	PWY-4821	17	18	4.23E-06	1.74E-04
Free phenylpropanoid acid biosynthesis	PWY-2181	9	9	4.10E-04	8.40E-03
Betanidin degradation	PWY-5461	64	106	9.06E-05	2.39E-03
Baicalein degradation (hydrogen peroxide detoxification)	PWY-7214	64	106	9.06E-05	2.39E-03
Luteolin triglucuronide degradation	PWY-7445	64	106	9.06E-05	2.39E-03
Homogalacturonan degradation	PWY-1081	32	49	8.21E-04	1.60E-02
Phenylpropanoids methylation (ice plant)	PWY-7498	15	19	1.18E-03	1.99E-02


*Bn (Background number), indicates the total number of unigenes present in each pathway. Nd (Number of DEGs), indicate the number of differentially expressed unigenes in each pathway.*

### Identification of Candidate Genes Involved in Oleanolic Acid and Ecdysterone Biosynthesis

**FIGURE 7:**
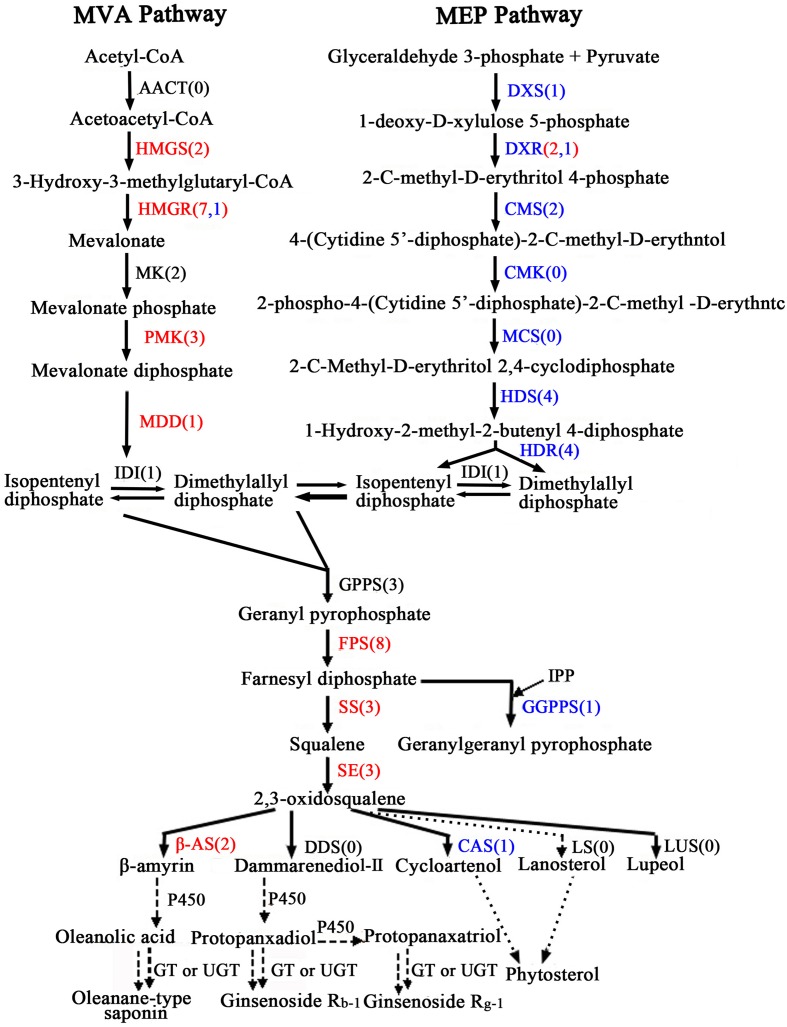
**Putative triterpenoid saponins biosynthesis pathway in *A. bidentata*.** Genes in red and blue mean up- and down-regulated in the roots as compared with leaves. Numbers in brackets represent numbers of unigenes in *A. bidentata*. AACT, acetyl-CoA acetyltransferase; HMGS, HMG-CoA synthase; HMGR, HMG-CoA reductase; MK, mevalonate kinase; PMK, phosphomevalonate kinase; MDD, mevalonate-5-diphosphate decarboxylase; DXS, 1-deoxy- D-xylulose 5-phosphate synthase; DXR, 1-deoxy-D-xylulose 5-phosphate reductoisomerase; CMS, 2-C- methyl-D-erythritol 4-phosphate cytidylyltransferase; CMK, 4-(cytidine 50- diphospho)-2-C-methyl-D-erythritol kinase; MCS, 2-C-methyl-D-erythritol-2,4- cyclodiphosphate synthase; HDS, 4-hydroxy-3-methylbut-2-en-1-yl diphosphate synthase; HDR, 4-hydroxy-3-methylbut-2-enyl diphosphate reductase; IDI, isopentenyl diphosphate isomerase; GPPS, geranyl diphosphate synthase; FPS, farnesyl diphosphatesynthase; GGPPS, geranylgeranyl diphosphate synthase; SS, squalene synthase; SE, squalene epoxidase; DDS, dammarenediol synthase; β-AS, beta-amyrin synthase; CAS, cycloartenol synthase; LS, lanosterol synthase; LUS, lupeol synthesis; P450, cytochrome P450; GT, glycosyltransferase; UGT, UDP-glycosyltransferase.

Oleanolic acid and ecdysterone are isoprenoid-derived compounds using the five-carbon building unit isopentenyl diphosphate (IPP) and its isomer dimethylallyl diphosphate (DMAPP). Unlike other living organisms, plant isoprenoid precursors are synthesized by two independent pathways: the mevalonate (MVA) pathway in the cytosol, and the non-MVA methylerythritol phosphate (MEP) pathway in plastids (Bach, 1995; Hemmerlin et al., 2012; Vranová et al., 2013), with some crosstalk between them (Hemmerlin et al., 2003; Laule et al., 2003). The former is responsible for the synthesis of sterols, certain sesquiterpenes, terpenoid aldehydes, triterpenoid saponins and the side chain of ubiquinone; in contrast, the recently discovered MVA-independent pathway is involved in providing the precursors for monoterpenes, certain sesquiterpenes, diterpenes, carotenoids, and the side chains of phytol, chlorophylls and plastoquinone (Harborne, 1991; Rohmer, 1999; Opitz et al., 2014). As far as the triterpenoid saponions are concerned, triterpenoid saponions are biosynthesized via different cyclization reactions of squalene. Squalene synthesis is the branch point of the central isoprenoid pathway and occurs at the triterpenoid saponin biosynthetic branch (Chappell, 1995; Chen et al., 2000; Hao et al., 2002). 2,3-oxidosqualene cyclases (OSCs), the rate-limiting enzymes in triterpenoid saponin and sterol biosynthesis branches, catalyze 2,3-oxidosqualene, which results in the formation of the triterpenoid skeleton, cycloartenol, and other compounds. Further study of cyclization reactions involving squalene reveals that 2,3-oxidosqualene is converted to cycloartenol by cycloartenol synthase (CAS) and lanosterol by lanosterol synthase (LS), while the molecule 2,3-oxidosqualene is converted to β-amyrin through β-amyrin synthase (β-AS). With further modifications, these compounds can then form a variety of triterpenoid saponins, phytosterols, and other macromolecules. The putative pathways of active components biosynthesis were shown in **Figure 7** according to our RNA-seq. Notably, we identified 260 (*P* < 0.05) unigenes encoding almost all the recognized enzymes which involve in oleanolic acid and ecdysterone biosynthesis via the MVA pathway (Supplementary Table S6). Moreover, 242 DEGs between the roots and leaves, with 122 up-regulated and 120 down-regulated genes, were identified. In addition, we also identified 14 DEGs encoding enzymes which involve in isoprenoid biosynthesis via MEP pathway (**Figure 7**; Supplementary Table S6).

**FIGURE 8:**
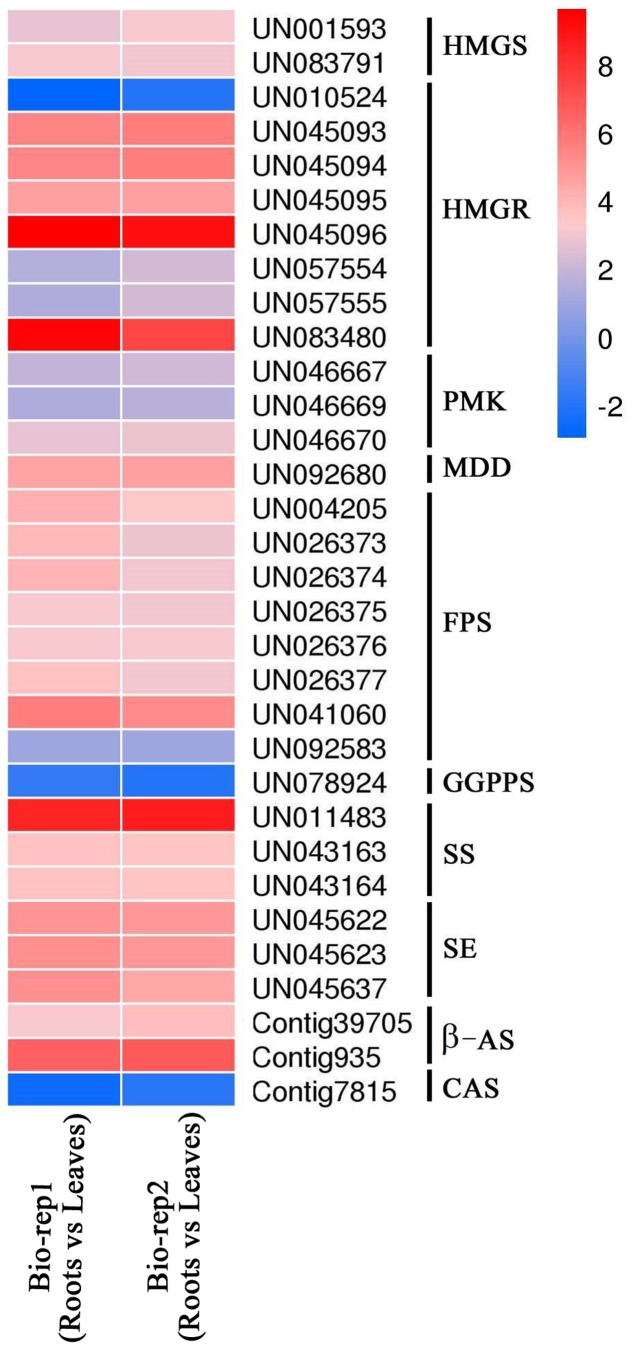
**Expression profile for the candidate unigenes from teiterpenoid saponins biosynthetic pathways across two tissues.** The bar represents the scale of the expression levels for each gene of roots vs. leaves as indicated by red/blue rectangles. Red indicates up-regulation of genes and blue indicates down-regulation of genes. Expression ratios are expressed as log2Fold Change (root/leaf).

In present study, multiple transcripts which encoded some of the known enzymes participated in the MVA pathway and the saponin biosynthesis pathway were found in traditional herbs, e.g., *Panax ginseng* (Cao et al., 2015), *Phyllanthus amarus* (Mazumdar and Chattopadhyay, 2016), *Panax japonicus* (Rai et al., 2016), *Astragalus membranaceus* Bge (Chen et al., 2015), and *Eleutherococcus senticosus* (Hwang et al., 2015). Genes involved in the MVA pathway in the *A. bidentata* transcriptome included 3-hydroxy-3-methylglutaryl CoA synthase (HMGS), HMG-CoA reductase (HMGR), phosphomevalonate kinase (PMK), mevalonate-5-diphosphate decarboxylase (MDD), isopentenyl diphosphate isomerase (IDI), geranyl diphosphate synthase (GPPS), farnesyl diphosphate synthase (FPS), geranylgeranyl diphosphate synthase (GGPPS), squalene synthase (SS), squalene epoxidase(SE), beta-amyrin synthase (β-AS), and CAS. The expression profiles of these unigenes were represented in the heat map which displayed the differential gene expression across various tissues. Most of the genes involved in triterpene saponins biosynthesis showed higher expression in root tissues compared with leaf tissues (**Figure 8**). Additionally, more experiments showed that genes involved in pathway of saponion biosynthesis displayed similar expression pattern in *Panax japonicus* (Rai et al., 2016), and *Astragalus membranaceus* Bge (Chen et al., 2015). However, the CAS gene was significantly down-regulated in root tissues. Previous reports have shown high ecdysterone accumulation in the leaves of *A. bidentata* at various stages, followed by the stem and root tissues (Hu, 2014). This finding is consistent with the CAS gene transcriptional level. In addition, more studies suggested that HMG-CoA reductase was considered as the first rate-limiting enzyme in the upstream biosynthetic pathway of triterpenes. According to homology and similarity analysis, we identified 3 (UN045093, UN045094, and UN045095) promising *HMGR* candidates out of 10 *HMGR* unigenes and as shown in **Supplementary Figure S1**. Sequence analysis showed that there were a very high similarity between UN045093, UN045094, UN045095 and the *HMGR1* of *Beta vulgaris*. So, they were most likely to be involved in the biosynthesis of triterpenes. And the range of their expression ratios (Root/Leaf) was from 26.35 to 48.09, it indicated that the rate of saponin biosynthesis in root was higher than in leaf of *A. bidentata*.

Following cyclization, the triterpene backbones undergo a series of (mainly) oxidative modifications at multiple carbon positions to generate triterpenoids (Moses et al., 2014). However, little is known about the modification reaction after cyclization in saponin biosynthesis. More recent studies have shown that some cytochrome P450 monooxygenases (CYP450s) and glycosyl transferases (GTs) are assumed to perform modifications on triterpenoid skeletons, including hydroxylation and glycosidation, which leads to the generation of various triterpenoids (Chen et al., 2015). These enzymes exist as supergene families in the plant genome. Additionally, recent experiments demonstrated that the CYP93E9 from *Phaseolus vulgaris* showed the highest activity and converted ∼80% of the accumulating *in vivo*-produced substrate β-amyrin into the products olean-12-ene-3β, 24-diol and likely 3β-hydroxy olean-12-en-24-oic acid with a catalytic efficiency that was 61 times higher than that of the *Medicago truncatula* CYP93E2 (Moses et al., 2014). In addition, the cytochrome P450 CYP716A52v2 has been illustrated to engage in the formation of oleanane-type ginsenoside biosynthesis in *P. ginseng* (Han et al., 2012, 2013). Saponins are high-molecular-weight glycosides consisting of a sugar moiety linked to a triterpenoid or a steroid aglycone, and plant UDP-glycosyltransferases (UGTs) are a widely divergent group of enzymes that transfer a sugar residue from an activated donor to an acceptor molecule (Chen et al., 2014). Moreover, it has been previously reported that UGT73 family genes participate in saponin glycosylation in other plants (Thimmappa et al., 2014). A total of 235 *UGTs* identified in the *P. ginseng* root transcriptome have previously been identified (Chen et al., 2011). However, in *A. bidentata*, the different functions of CYP450 and GT enzymes which involved in saponin biosynthesis remain unknown. In present study, we identified a total of 115 *P450* unigenes, of which 48 genes were up-regulated, 59 genes were down-regulated and eight candidate genes did not show any differential expression in the roots compared with the leaves. Moreover, we identified 92 *UGT* unigenes (28 up-regulated and 58 down-regulated) and 17 *GTs* (16 up-regulated), as shown in Supplementary Table S6.

### Identification of Candidate Genes Involved in Saponin Transport

Compared with the biosynthesis, the transport and regulation of saponin in cells remains unknown. Therefore, our study aimed to unravel the principal genes participating in the transport of secondary metabolites through plant pleiotropic drug resistance (PDR) transporter, a ATP-binding cassette (ABC) transporter from ABCG subfamily. The family of full-length ABC transporters can be subdivided into four major subfamilies: pleiotropic drug resistance (PDR), multidrug resistance (MDR), MDR-associated protein (MRP), and ABCA (van den Brûle and Smart, 2002; Rea, 2007). In the present study, the identification and expression of many ABC members, such as *MDR* and *PDR* genes, were screened in the transcriptome dataset. Among the unigene hits, a total of 85 unigenes for ABC (from ABCA to ABCG), 23 unigenes for MDR, and 8 unigenes for PDR were identified (**Table 4**; Supplementary Table S7). Further evidence demonstrated that PDR transporters are involved in exporting a wide range of substrates across membranes in various biological processes, including defense against pathogens and salinity stresses, secondary metabolites and plant hormone transportation (Kim et al., 2010; Bienert et al., 2012; Kretzschmar et al., 2012). In *P. ginseng*, uptake experiments demonstrated a potential role of the *PgPDR3* gene in the accumulation of secondary metabolites in MeJA-induced ginseng (Zhang et al., 2013), and *NtPDR1* is involved in plant defense through diterpene transport in *Nicotiana tabacum* (Crouzet et al., 2013). In the present study, we also identified 8 *PDR* unigenes, 2 of which were up-regulated, in the root compared with the leaf.

**Table 4:** Numbers of ABC transporters involved in terpenoid biosynthesis pathways identified putatively from *A. bidentata* transcriptome.

Name of enzymes	Number of unigenes	Up-regulated	Down-regulated	Not DEGs
MDR	23	13	8	2
PDR	8	2	4	2
ABC	54	23	24	7


### Real-Time Quantitative PCR Validation of RNA-Seq Results

**FIGURE 9:**
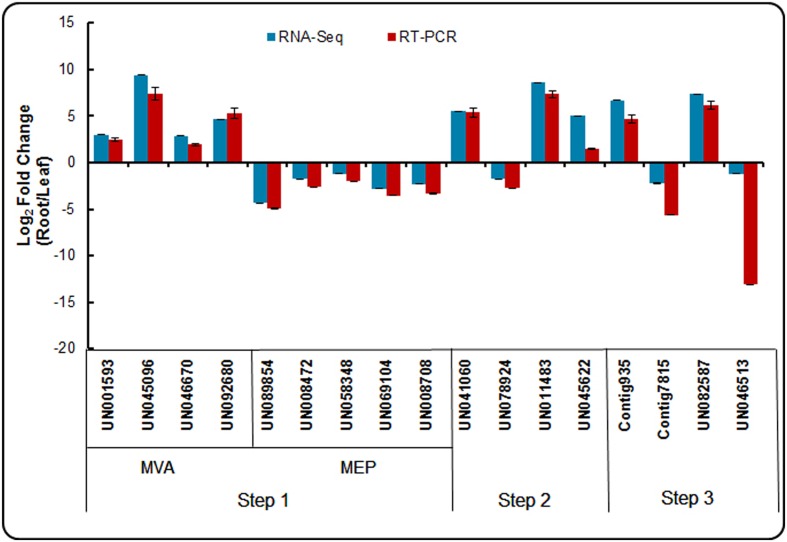
**Expression patterns of genes involved in triterpenoid saponion and sterol biosynthesis pathway detected by RNA-seq and qRT-PCR**.

To confirm the reliability of the RNA-Seq data, an appropriate alternative methodology was selected. Seventeen DEGs participating in the triterpenoid saponin and ecdysterone biosynthesis pathways were selected for validation using real-time qPCR. The identification of crucial enzymes from our dataset involved from acetyl-CoA to saponin and ecdysterone biosynthesis are presented in three steps. And the expression patterns of these genes in the root and leaf are shown in **Figure 9**. The expression patterns determined using real-time qPCR results demonstrating the expression patterns of these genes were consistent with RNA-Seq. It confirmed the accuracy of the RNA-Seq results in this study.

## Discussion

*Achyranthes bidentata* has long been used as an herbal medicine. However, little genomic information is available for this species. At present, there is one report of transcriptome studies on bidentata species using 454 pyrosequencing combined with magnetic bead enrichment (Li et al., 2015). With the development of large scale genomics, high-throughput Illumina/Solexa sequencing technology has recently been a fast, efficient, inexpensive, accurate and reliable tool for transcriptome characterization and gene discovery in non-model organisms as well. In the present study, we employed RNA-Seq technology on various *A. bidentata* tissue samples (root and leaf) obtained during vegetative growth. Bidentata *de novo* transcriptome assembly resulted in 100,987 unigenes with a mean length of 1146.8 bp and total residues of 115,810,705 bp. GO, COG, and KEGG pathway mapping identified 31,634 of these high-quality unigenes as putative homologs of annotated sequence in the protein databases. Among the total transcripts, 20.29% (20,488/100,987), 21.50% (21,715/100,987), and 12.63% (12,762/100,987) were annotated based on their homologs using the GO, COG, and KEGG pathway databases, respectively. Using this information, we identified and investigated *A. bidentata* genes related to oleanane-type saponin and ecdysterone biosynthesis. These data provide a valuable resource for breeding and further research, particularly in studies of the biosynthesis pathways for the pharmacologically active components.

Oleanolic acid and ecdysterone, as pharmacologically effective metabolites, are two of the major extractions from *A. bidentata*. As we know, triterpene saponins are synthesized through the MVA biosynthetic pathway and are one of the most important classes of natural products, of which glycoside conjugation has important medicinal properties. However, we also identified some enzymes from the non-MVA (MEP/DOXP) pathway involved in biosynthesis of saponins (**Figure 7**). 2-C-methyl-D-erythritol-4-phosphate (MEP) operates inside the plastid compartment, and it is initiated by a transketolase catalyzed condensation of glyceraldehyde 3-phosphate (GAP) with pyruvate. In our study, genes encoding isoprenoids biosynthetic enzymes of non-MVA pathway were found including 1-deoxy-D-xylulose 5-phosphate synthase (DXS), 1-deoxy-D-xylulose 5-phosphate reductoisomerase (DXR), 2-C-methyl-D-erythritol 4-phosphate cytidylyltransferase (CMS), 4-hydroxy-3-methylbut-2-en-1-yl diphosphate synthase (HDS) and 4-hydroxy-3-methylbut-2-enyl diphosphate reductase (HDR), as shown in **Figure 7** and Supplementary Table S6. Moreover, most of these enzymes displayed higher expression in the leaves than in the roots. The results are consistent with the expression of genes which involve in triterpenoid saponins biosynthesis in various organs of *Astragalus membranaceus* Bge (Chen et al., 2015). It clearly demonstrates that non-MVA pathway is the choice for the leaf, whereas MVA pathway is preferred in the root. Moreover, More recent studies revealed that 2-*C*-methyl-D-erythritol 4-phosphate cytidyltransferase (CMS), DXS, HDS, and DXR are highly expressed in young photosynthetic tissues and flower organs of *Arabidopsis* at the protein level (Estevez et al., 2000; Carretero-Paulet et al., 2002; Gutierrez-Nava et al., 2004). This finding suggested that the regulation of the isoprenoid biosynthesis pathway is different in plant organs.

Crosstalk of between the plastidial and cytosolic pathways in *Ginkgo biloba* seedlings and *Catharanthus roseus* cells with stable isotopes had been confirmed by preceding scholars (Schwarz, 1994; Arigoni et al., 1997). This behavior suggested a potential exchange of isoprenoids between the plastid and the cytosol. Moreover, plastid membranes possess a unidirectional proton symport system for the export of specific isoprenoid intermediates involved in the metabolic cross talk between the cytosolic and plastidial pathways of isoprenoid biosynthesis in *Arabidopsis thaliana* (Laule et al., 2003). Furthermore, there is no absolute compartmental separation of the two pathways, and the extent of this cross-talk depends on the species and the physiological conditions (Luan and Wüst, 2002; Hemmerlin et al., 2003). Similarly, feeding experiments also indicated that in *Arabidopsis*, ent-kaurene and campesterol derived from 1-deoxy-D-xylulose (68–87%) and (3–7%), respectively, and from MVA (5–8%) and (80%), respectively (Kasahara et al., 2002). In leaves and roots of carrot, although monoterpenes were synthesized exclusively via the DXP pathway, the sesquiterpenes derived from the MVA and the DXP pathways (Hampel et al., 2005). Thus, ecdysterone and oleanolic acid are synthesized mainly through the MVA pathway, providing specific isoprenoid intermediates via the MEP pathway. Previous reports of *A. bidentata* had shown the highest ecdysterone accumulation in the leaves of at various stages, followed by the stem and root tissues (Hu, 2014). In this study, compared with the roots, except for the CAS gene, which involved in MVA pathway, were significantly down-regulated in the leaves of *A. bidentata*. Contrarily, most of the genes involved in MEP pathway displayed higher expression in the leaves than in the roots. It might be because that CAS gene which involved in catalyzed 2,3-oxidosqualene converted to cycloartenol displayed higher expression in the leaves compared with the roots, and the β-amyrin synthase (β-AS) gene belong to OSC famliy genes expressed lowly in the leaves. In addition, some isoprenoids and other isoprenoid intermediates in plastid can be transfer into cytosol in order to participate in the production of sesquiterpenes, triterpenes, sterols, etc. So we speculate that ecdysterone might be synthesized mainly in leaves of *A. bidentata*. These findings will provide a theoretical basis for improving the contents of active ingredients in medicinal plants.

Eecdysterone of *A. bidentata* extracts markedly increases osteoblastic activity and protects chondrocytes (Gao et al., 2000; Zhang et al., 2014). However, content of edysterone of leaves and roots is very low in *A. bidentata*, only 0.555 mg/g and 0.222 mg/g, respectively (Hu, 2014). Now, the yield of *A. bidentata* is seriously reduced by the disease and insect pest, but the improvement in yield and quality of *A. bidentata* is only limited to the control of field management at present. Along with the rapid development and extensive application of molecular biology, genetic engineering has become an effective approach to enhance the contents of active ingredients in medicinal plants. In present study, putative genes involved in the biosynthetic pathway of phytoecdysteroid and modification of backbone had been identified. These findings will provide a theoretical basis for improving the contents of active ingredients in medicinal plants and thereby promote the development and utilization of *A. bidentata*.

These experiments also demonstrated that the biosynthesis of saponins, such as oleanane-type saponins, involves a series of key enzymes, including the encoded products of SS and β-AS, synthesized via a complex pathway. At the subcellular level, saponins are accumulated in vacuoles (Han et al., 2006; Mylona et al., 2008). However, OSCs, P450s and some UGTs involved in saponin biosynthesis are microsomal enzymes (Kurosawa et al., 2002), suggesting the presence of a vacuolar transporter of saponin. When plants are subjected to environmental stresses, secondary metabolites are synthesized and transported out of the cells (Yazaki, 2006; Yazaki et al., 2008). A recent study reported that the *Petunia hybrida* ABC transporter PDR1 acts as a cellular strigolactone exporter in regulating the development of arbuscular mycorrhizae and axillary branches (Kretzschmar et al., 2012), and some ABC transporters, including yeast PDR transporters, transport various secondary metabolites that function in detoxification processes (Crouzet et al., 2013). Indirect evidence suggests that NpPDR1 transports sclareol (Jasiński et al., 2001; Stukkens et al., 2005), and AtPDR12 and SpTUR2 also seem to be involved in sclareol transport (van den Brûle and Smart, 2002; Campbell et al., 2003). These results suggest that transporters for secondary metabolites are important to plants. Our former studies suggested that oleanolic acid content of root increased gradually again, while oleanolic acid content of stem and leaf began to decrease gradually during late stage of reproductive growth (Li and Hu, 2009). In this study, we also found the expression level of 4 PDRs in the leaf were higher than that in the root. The reason may be that transporters are involved in the transport of secondary metabolites from leaves to roots. Thus, it would be promising in regulation some useful secondary metabolites biosynthesis and accumulation by using the *PDR* genes. Moreover, improving the accumulation by engineering transport systems might be an attractive strategy to increase the production of secondary metabolites for the drug industry.

## Conclusion

This study revealed the *de novo* transcriptome sequencing analysis of *A. bidentata* for the first time by utilizing Illumina RNA-Seq. In total, 100,987 unigenes were assembled, and 31,614 sequences were annotated, including 12,762 unigenes mapped to 303 pathways. In particular, a number of the unigenes involved in oleanane-type saponin and ecdysterone synthesis pathways were identified according to GO analysis and KEGG assignment. The identified sequences associated with saponin biosynthesis will facilitate the study of the functional genomics of saponin biosynthesis and the genetic engineering of *A. bidentata*. Further qRT-PCR results indicating expression of the few chosen unigenes participated in bioactive component synthesis also confirmed the reliability and accuracy of our *A. bidentata* tanscriptome assembly. This report will provide valuable information for future studies on herbs, greatly boosting study on no-model plants.

## Deposited Data

The Illumina RNA-sequencing raw reads of *A. bidentata* are available from the NCBI Sequence Read Archive database (http://www.ncbi.nlm.nih.gov/sra/) under project Accession number of PRJNA350183.

## Author Contributions

CW, XH, WQ, YC, and TW performed the sample collection, analyzed the data, and wrote the manuscript. YZ performed the sequence analysis. XZ and JL designed the experiment, revised the manuscript, and provided guidance on the whole study. All authors have read and approved the manuscript.

## Conflict of Interest Statement

The authors declare that the research was conducted in the absence of any commercial or financial relationships that could be construed as a potential conflict of interest.

The reviewer LR and handling Editor declared their shared affiliation, and the handling Editor states that the process nevertheless met the standards of a fair and objective review.

## Footnotes

1 http://nar.oxfordjournals.org/content/43/D1/D1049

2 http://search.cpan.org/dist/GO-TermFinder/

## Supplementary Material

**FIGURE S1 | Phylogenetic analysis of five identified HMGR from our dataset and other plant HMGRs.** Phylogenetic tree was generated using the neighbor-joining (NJ) method in MEGA6. HMGR of this study is shown in red box.
