# Supplementary figures and images for: Transcriptome Analysis to Identify the Putative Biosynthesis and Transport Genes Associated with the Medicinal Components of Achyranthes bidentata Bl
Source: Front Plant Sci. 2016 Dec 12;7:1860. doi: 10.3389/fpls.2016.01860 (PMC5149546; doi:10.3389/fpls.2016.01860)

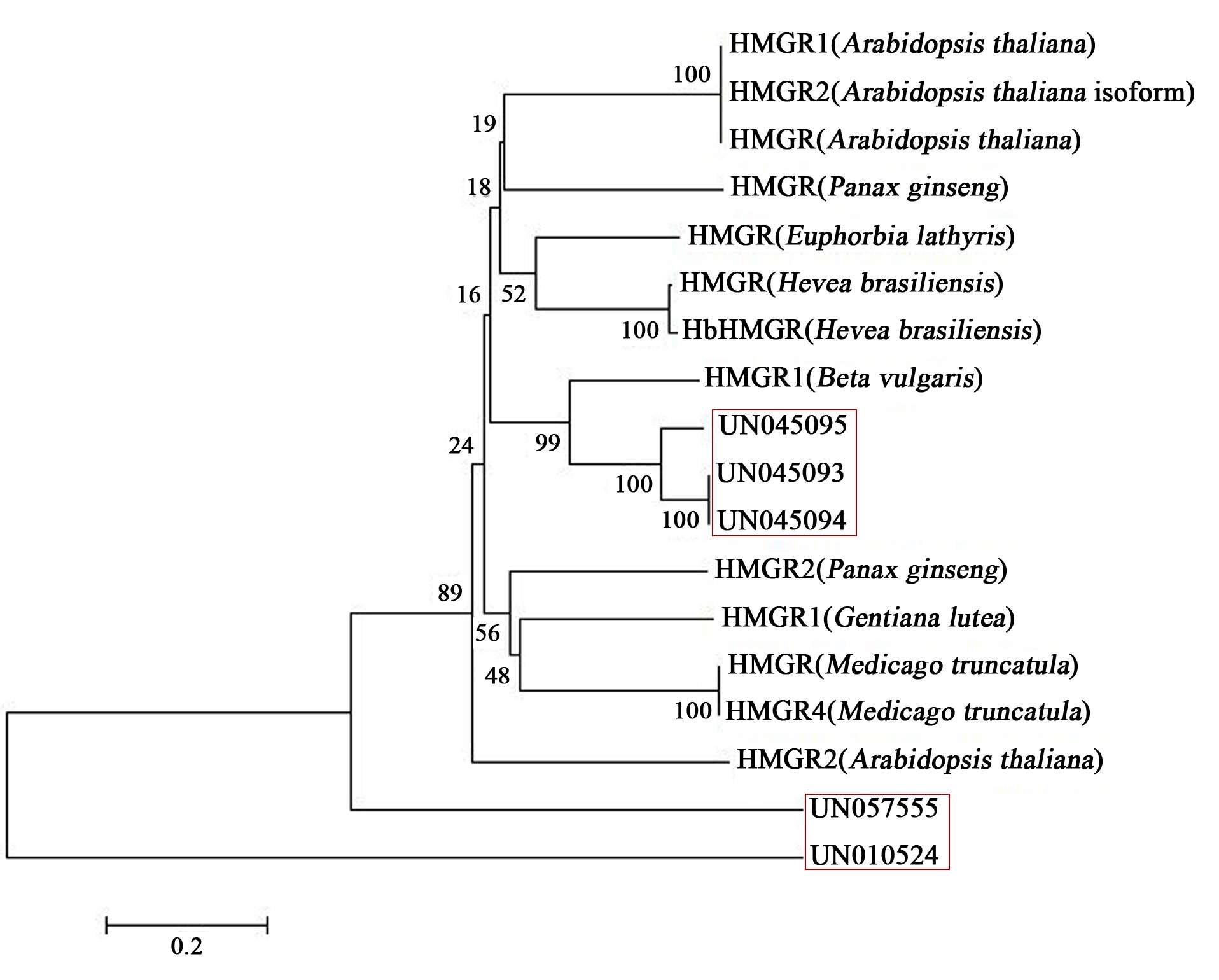

Supplement: Supplementary file 1 [file Image_1.JPEG]
