# Supplementary material for: Transcriptome Analysis to Identify the Putative Biosynthesis and Transport Genes Associated with the Medicinal Components of Achyranthes bidentata Bl
Source: Front Plant Sci. 2016 Dec 12;7:1860. doi: 10.3389/fpls.2016.01860 (PMC5149546; doi:10.3389/fpls.2016.01860)
Supplement: Supplementary file 2 [file Table_1.DOCX]

**Supplementary Table S1: Primer information of unique transcripts for the qRT-PCR analysis**

| **Accession No.** | **Annotation** | **Primer set** | |
| --- | --- | --- | --- |
|  |  | **Forward primer (5’-3’)** | **Reverse primer (5’-3’)** |
| UN001593 | HMGS1 | GGCAAGAGGGTCATACTGT | TTCTCAGGTGGGAACTCAT |
| UN045096 | HMGR5 | ACGCCGCCATTGTCACTTT | CAAGACGATGAGCGAGGGG |
| UN046670 | PMK3 | TGGGTTGGGTTCCTCTGC | CTTTCCCTTGGGCGATACA |
| UN092680 | MDD | CATAGCGAGGCAAGGTTCTG | CTCACGCATCCCACTAGTACTG |
| UN041060 | FPS7 | GATACTTCTCGTCAATGGGTT | CTTCTCCGTCCTTCAGCA |
| UN078924 | GGPPS | ATAGCACAGACCGTGGGATC | CACCAGCCAATAAGCCTCC |
| UN011483 | SS1 | GGTTCAACTTGCTGCCTTTA | ATTGCCACCTGGTAACTGC |
| UN045622 | SE1 | GCAGGTGCTTTATACAAGGT | CAATGGTTTAGGGTTCAGG |
| Contig935 | beta-AS1 | CCTCAAGTGTTGCCTAAT | GCCCTCCTTTCTTACTCT |
| Contig7815 | CAS | AGATGTTGAGGGAGAAGGCG | GGTCTTCCACCCAACAACAAA |
| UN082587 | CYP450-98 | CTCCACTCCAAGGCGGTT | AGGTTCTGGGAAGCAATCTG |
| UN046513 | UGT51 | ATTGCCTGATGGGTTTGA | ACTGGCTTTCCAACCTTTA |
| UN089854 | DXS | GGGTTGTCAGGGTTTACG | TGGCACCATCACCTATAACA |
| UN008472 | DXR1 | GCGATCTTCCAGTGTATCC | TGCTCCAGTTAGGGTGTTT |
| UN058348 | CMS1 | CTACCGCTGAACTTGTCTGC | GCTCCGTGTATCAATCCATCT |
| UN069104 | HDS4 | ATAAGAGGGAAGACATCACAC | CAGGAGAACCACCAACATA |
| UN008708 | HDR1 | GATGCTACTCAAGAGCGACAAG | GCTCCTCGGCTATCTCCTG |
| UN008343 | UBI | AGAGGTTGATGCGGGATTTC | GATAACGGCATTCCAGAGCA |
